# Invalidation of dieckol and 1,2,3,4,6-pentagalloylglucose (PGG) as SARS-CoV-2 main protease inhibitors and the discovery of PGG as a papain-like protease inhibitor

**DOI:** 10.21203/rs.3.rs-1490282/v1

**Published:** 2022-03-30

**Authors:** Haozhou Tan, Chunlong Ma, Jun Wang

**Affiliations:** Rutgers University New Brunswick; University of Arizona College of Pharmacy: The University of Arizona College of Medicine Phoenix; Rutgers The State University of New Jersey

**Keywords:** SARS-CoV-2, main protease, papain-like protease, antiviral, coronavirus

## Abstract

The COVID-19 pandemic spurred a broad interest in antiviral drug discovery. The SARS-CoV-2 main protease (M^pro^) and papain-like protease (PL^pro^) are attractive antiviral drug targets given their vital roles in viral replication and modulation of host immune response. Structurally disparate compounds were reported as M^pro^ and PL^pro^ inhibitors from either drug repurposing or rational design. Two polyphenols dieckol and 1,2,3,4,6-pentagalloylglucose (PGG) were recently reported as SARS-CoV-2 main protease (M^pro^) inhibitors. With our continuous interest in studying the mechanism of inhibition and resistance of M^pro^ inhibitors, we report herein our independent validation/invalidation of these two natural products. Our FRET-based enzymatic assay showed that neither dieckol nor PGG inhibited SARS-CoV-2 M^pro^ (IC_50_ > 20 μM), which is in contrary to previous reports. Serendipitously, PGG was found to inhibit the SARS-CoV-2 papain-like protease (PL^pro^) with an IC_50_ of 3.90 μM. The binding of PGG to PL^pro^ was further confirmed in the thermal shift assay. However, PGG was cytotoxic in 293T-ACE2 cells (CC_50_ = 7.7 μM), so its intracellular PL^pro^ inhibitory activity could not be quantified by the cell-based Flip-GFP PL^pro^ assay. In addition, we also invalidated ebselen, disulfiram, carmofur, PX12, and tideglusib as SARS-CoV-2 PL^pro^ inhibitors using the Flip-GFP assay. Overall, our results call for stringent hit validation, and the serendipitous discovery of PGG as a putative PL^pro^ inhibitor might worth further pursuing.

## Introduction

COVID-19 is caused by the SARS-CoV-2, an enveloped, single-stranded, and positive-sense RNA virus [1]. Seven coronaviruses are known to infect humans including four common human coronaviruses OC43, 229E, NL63, and HKU1, and three highly pathogenic coronaviruses SARS-CoV, SARS-CoV-2 and MERS-CoV [2]. The COVID-19 pandemic is a timely call for the urgent need of orally bioavailable antivirals. Drug repurposing plays a pivotal role in advancing drug candidates to clinic [3]. For example, the first FDA-approved COVID drug, remdesivir, was originally developed for Ebola virus [4], and was later found to have broad-spectrum antiviral activity against several viruses including SARS-CoV, MERS-CoV, and SARS-CoV-2 [5, 6]. Similarly, molnupiravir was a clinical candidate for the influenza virus before repurposed for SARS-CoV-2 [7, 8]. The SARS-CoV-2 main protease (M^pro^) and papain-like protease (PL^pro^) are also high-profile drug targets for drug repurposing. Numerous virtual screenings and high-throughput screenings have been conducted, revealing structurally disparate inhibitors that are at different stages of preclinical and clinical development [9]. For example, boceprevir [10, 11], calpain inhibitors [10], GC-376 [10, 12], and masitinib [13] were among the first hits reported as M^pro^ inhibitors. GRL0617 [14, 15], YM155 [16], 6-thioguanine [17], SJB2-043 [18], and others were identified as PL^pro^ inhibitors. Natural products are also a rich source of modern medicine [19], and multiple natural products have been reported as M^pro^ and PL^pro^ inhibitors [20]. For example, two polyphenols dieckol and 1,2,3,4,6-pentagalloylglucose (PGG) were recently reported as SARS-CoV-2 main protease (M^pro^) inhibitors [21, 22]. With our continuous interest in validation/invalidation of literature reported SARS-CoV-2 M^pro^ and PL^pro^ inhibitors [23–26], we report herein our independent validation of these two compounds using the established FRET enzymatic assay and cell-based Flip-GFP assay. In addition, we further confirmed that the previously reported promiscuous cysteine modifiers ebselen, disulfiram, carmofur, PX12, and tideglusib [27] are not PL^pro^ inhibitors, despite the claim from several publications that they act as PL^pro^ inhibitors [28, 29]. Interestingly, we serendipitously discovered PGG as a PL^pro^ inhibitor and showed that PGG binds to PL^pro^ and inhibited the enzymatic activity of PLpro in the FRET assay. Taken together, our results call for stringent hit validation, and the serendipitous discovery of PGG as a putative PL^pro^ inhibitor might worth further investigation.

## Results And Discussion

### Invalidation of dieckol and PGG as SARS-CoV-2 M ^pro^ inhibitors.

Dieckol was reported as a SARS-CoV-2 M^pro^ inhibitor through a fluorescence polarization-based high-throughput screening [21]. In the assay design, the biotin-labeled M^pro^ substrate was conjugated with a fluorescein isocyanate (FITC) fluorophore, resulting in a bifunctional probe FITC-AVLQ↓SGFRKK-Biotin (FITC-S-Biotin). Binding of this probe to avidin led to increased fluorescence polarization. Upon M^pro^ digestion, the fluorophore-peptide conjugate FITC-AVLQ was released, which correlates with reduced millipolarization unit (mP) signal. Screening of a natural product library of 5,000 compounds identified dieckol as a potent M^pro^ inhibitor with IC_50_ values of 4.5 μM (no DTT) and 2.9 μM (1 mM DTT). The mechanism of action was characterized using the FRET assay and surface plasmon resonance binding assay, both of which showed consistent results as the FP assay. Enzymatic kinetic studies demonstrated that dieckol is a competitive M^pro^ inhibitor. It is noted that dieckol was also previously reported as a SARS-CoV M^pro^ inhibitor [30].

PGG was reported as an inhibitor for both SARS-CoV and SARS-CoV-2 M^pro^ with IC_50_ values of 6.89 and 3.66 μM, respectively [22]. In another study, PGG was found to bind to the SARS-CoV-2 spike protein receptor binding domain (RBD) with a K_D_ of 6.69 μM in the bio-layer interferometry assay, while the binding of PGG to the ACE2 receptor was weaker with a K_D_ of 22.2 μM [31]. PGG was further shown to block the RBD-ACE2 interactions in the ELISA assay with an IC_50_ of 46.9 μM. In the SARS-CoV-2 pseudovirus assay, PGG dose-dependently inhibited the viral entry and replication.

**Figure 1:**
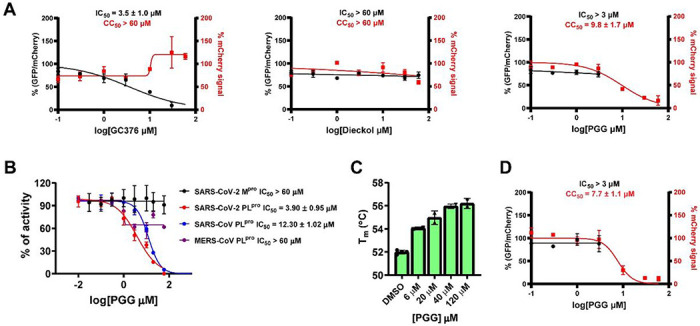
Validation and invalidation of dieckol and PGG as SARS-CoV-2 M^pro^ and PL^pro^ inhibitors. (A) Flip-GFP M^pro^ assay results of dieckol and PGG. GC376 was included as a positive control. (B) FRET assay results of PGG against SARS-CoV-2 M^pro^, SARS-CoV-2 PL^pro^, SARS-CoV PL^pro^, and MERS-CoV PL^pro^. (C) Thermal shift assay characterization of the binding of PGG to SARS-CoV-2 PL^pro^. (D) Flip-GFP PL^pro^ assay result of PGG. The results are mean ± standard deviation of two repeats.

To validate whether dieckol and PGG are M^pro^ inhibitors, we repeated the FRET enzymatic assay using our standard FRET assay condition (20 mM HEPES, pH 6.5, 120 mM NaCl, 0.4 mM EDTA, 4 mM DTT, and 20% glycerol). Both dieckol and PGG were inactive (IC_50_ > 20 μM) (Table 1). To examine whether dieckol and PGG inhibited the intracellular protease activity of M^pro^, we characterized both compounds in the cell-based Flip-GFP M^pro^ assay. Our previous results showed that there is generally a positive correlation between the Flip-GFP and antiviral assay results, while the correlation between the FRET enzymatic assay results and antiviral assay results is compound dependent [15]. In the Flip-GFP assay, the GFP is reconstituted upon cleavage of the engineered linker by M^pro^, and the normalized GFP/mCherry signal ratio is proportional to the M^pro^ activity (mCherry serves as an internal control for the protein expression level or compound toxicity) [32, 33]. GC-376 was included as a positive control and it showed an EC_50_ of 3.5 μM (Fig. 1A). The results showed that both compounds lacked the cellular M^pro^ inhibitory activity at non-toxic drug concentrations (Fig. 1A). Dieckol was not active (IC_50_ > 60 μM), while PGG was cytotoxic (CC_50_ = 9.8 μM) (Fig. 1A), therefore the result was not conclusive. Taken together, dieckol and PGG were both invalidated as M^pro^ inhibitors.

In parallel, we tested dieckol and PGG against SARS-CoV-2 PL^pro^ in the FRET assay. While dieckol was not active (IC_50_ > 20 μM), PGG was serendipitously found to inhibit SARS-CoV-2 PL^pro^ with an IC_50_ of 3.9 μM (Fig. 1B and Table 1). To profile the broad-spectrum activity, PGG was tested against SARS-CoV and MERS-CoV PL^pro^. PGG showed weak activity against SARS-CoV PL^pro^ with an IC_50_ of 12.3 μM, while it was inactive against the MERS-CoV (IC_50_ > 60 μM) (Fig. 1B). These results suggest that the inhibition of SARS-CoV-2 PL^pro^ by PGG might be specific. We further characterized the binding of PGG to SARS-CoV-2 PL^pro^ in the thermal shift assay and found that PGG increased the thermal stability of PL^pro^ in a dose dependent manner (Fig. 1C). To determine whether PGG inhibits the intracellular protease activity of SARS-CoV-2 PL^pro^, we performed the Flip-GFP PL^pro^ assay. Unfortunately, PGG was cytotoxic to the 293T cells used in the Flip-GFP PL^pro^ assay (CC_50_ = 7.7 μM), resulting in inconclusive results (Fig. 1D).

**Figure 2:**
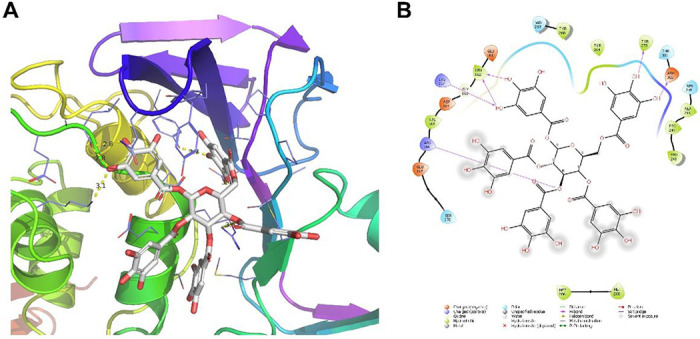
Docking model of PGG in SARS-CoV-2 PL^pro^. (A) Docking pose of PGG in the BL2 binding site of PL^pro^. (B) 2D ligand-protein interaction plot of PGG with SARS-CoV-2 PL^pro^. Docking was performed using the X-ray crystal structure of SARS-CoV-2 PL^pro^ (PDB; 7JRN). The Glide score was −10.024 from the Schrödinger Glide extra-precision docking.

To gain insights of the binding mode, we performed molecular docking of PGG with SARS-CoV-2 PL^pro^ (PDB: 7JRN) [15] using the Schrödinger Glide extra-precision. The binding sites in PL^pro^ were determined by the sitemap, which revealed the BL2 loop region as the highest-ranking binding site, therefore it was selected for PGG docking. The BL2 loop region is also the drug binding site of the known PL^pro^ inhibitors GRL0617 [15]. Docking results showed that PGG fits snugly in the binding site with a Glide score of −10.024 (Fig. 2A). PGG formed multiple hydrogen bonds with PL^pro^ residues including the side chains of Tyr273, Asp302, Arg166, Lys157 and the main chain of Leu162 (Fig. 2B).

### Invalidation of disulfiram, ebselen, carmofur, PX-12, and tideglusib as SARS-CoV-2 PL ^pro^ inhibitors.

Disulfiram was previously reported as a PL^pro^ inhibitor of both SARS-CoV and MERS-CoV [28]. Enzymatic kinetic studies showed that disulfiram acts as an allosteric inhibitor of MERS-CoV PL^pro^ and a competitive inhibitor of the SARS-CoV PL^pro^. In contrary, our previous study revealed that the inhibition of SARS-CoV-2 PL^pro^ by ebselen in the FRET-based enzymatic assay is reducing reagent dependent [25]. Ebselen inhibited SARS-CoV-2 PL^pro^ with an IC_50_ of 6.9 μM in the absence of DTT but was not active in the presence of DTT (IC_50_ > 60 μM) (Table 1). Likewise, ebselen, carmofur, PX-12, and tideglusib all showed various degrees of inhibition against the SARS-CoV-2 PL^pro^ in the absence of DTT, while the inhibition was abolished in the presence of DTT (Table 1) [25]. In contrary, Weglarz-Tomczak et al reported that ebselen inhibited SARS-CoV and SARS-CoV-2 PL^pro^s with IC_50_ values of 8.45 and 2.26 μM, respectively, in the presence of 2 mM DTT [29]. Disulfiram and ebselen were also proposed to inhibit SARS-CoV-2 PL^pro^ through ejecting zinc from the zinc-binding domain [34]. Given the debate whether reducing reagent should be added to the cysteine protease assay buffer, coupled with the controversy FRET assay results of ebselen in the presence of DTT, we were interested in further characterizing the inhibition of SARS-CoV-2 PL^pro^ by these compounds in a native cellular environment. For this, we employed our recently established cellular Flip-GFP PL^pro^ assay [15] to test the intracellular activity of these compounds. It was found that none of the compounds tested reduced the GFP/mCherry ratio at non-cytotoxic concentrations (Fig. 3), suggesting they lack the intracellular target engagement and PL^pro^ inhibition. Collectively, our data suggest that disulfiram, ebselen, carmofur, PX-12, and tideglusib should not be classified as PL^pro^ inhibitors.

## Conclusion

In conclusion, our data suggested that dieckol and PGG are not M^pro^ inhibitors as shown from the FRET and Flip-GFP M^pro^ assays. Furthermore, the previous reported promiscuous cysteine modifiers ebselen, disulfiram, carmofur, PX-12, and tideglusib were also invalidated as PL^pro^ inhibitors by the Flip-GFP PL^pro^ assay. Taken together with our previous efforts in invalidating these compounds as M^pro^ inhibitors, it can be concluded that M^pro^ and PL^pro^ enzymatic assay results obtained in the absence of reducing reagents have no correlation with their cellular activity. Among the list of compounds examined, ebselen was previously shown to inhibit SARS-CoV-2 viral replication in cell culture [27, 35]. Coupled with the results presented here, it appears that the antiviral mechanism of action of ebselen is independent of either M^pro^ or PL^pro^ inhibition.

Since the FRET assay conditions used in different labs vary, it might be challenging to directly compare the results. Nonetheless, the cell-based Flip-GFP assay is a valuable tool in evaluating the intracellular protease activity and is a close mimetic of virus-infected cells.

In summary, the results presented herein call for stringent hit validation before investing resources for lead optimization and translational antiviral development. The discovery of PGG as a PL^pro^ inhibitor provides another starting point for further optimization.

## Materials And Methods

All compounds were purchased from commercial source without further purification. PGG was ordered from Toronto Research Chemical with the Cat # P270450.

### SARS-CoV-2 M ^pro^ and PL^pro^ expression and purification.

SARS-CoV-2 main protease (M^pro^) gene from strain BetaCoV/Wuhan/WIV04/2019 (GenBank: MN996528.1) was purchased from GenScript (Piscataway, NJ) with E. coli codon optimization and inserted into pET29a(+) plasmid. The M^pro^ genes were then subcloned into the pE-SUMO plasmid as previously described [10, 36]. The expression and purification procedures were previously described [10]. SARS-CoV-2 papain-like protease (PL^pro^) gene (ORF 1ab 1564–1876) from strain BetaCoV/Wuhan/WIV04/2019 with *Escherichia coli* codon optimization was ordered from GenScript in the pET28b(+) vector. The detailed expression and purification procedures were previously described [15].

### FRET-Based Enzymatic Assay.

For the IC_50_ measurement with the FRET-based assay, the reaction was carried out in 96-well format with 100 μL of 200 nM PL^pro^ protein in a PL^pro^ reaction buffer (50 mM HEPES (pH 7.5), 5 mM DTT, and 0.01 % Triton X-100); 1 μL of testing compounds at various concentrations was added to each well and was incubated at 30°C for 30 min. The reaction was initiated by adding 1 μL of 1 mM FRET substrate and was monitored in a Cytation 5 image reader with filters for excitation at 360/40 nm and emission at 460/40 nm at 30°C for 1 h. The initial velocity of the enzymatic reaction was calculated from the initial 10 min enzymatic reaction. The IC_50_ was calculated by plotting the initial velocity against various concentrations of testing compounds using a four-parameter variable slope dose – response curve in Prism 8 software. IC_50_ values for the testing compounds against SARS-CoV-2 M^pro^ was determined as previously described [10].

### Flip-GFP M ^pro^ and PL^pro^ assay.

Plasmid pcDNA3-TEV-FlipGFP-T2A-mCherry was ordered from Addgene (catalog No.124429). pcDNA3 FlipGFP-M^pro^ plasmid and pcDNA3 FlipGFP-PL^pro^ plasmid were constructed by introducing SARS-CoV-2 M^pro^ cleavage site AVLQSGFR and SARS-CoV-2 PL^pro^ cleavage site LRGGAPTK, respectively, via overlapping PCRs. pLVX SARS-CoV-2 M^pro^ and pcDNA3.1 SARS-CoV-2 PL^pro^ plasmids was ordered from Genescript (Piscataway NJ) with codon optimization.

The Flip-GFP M^pro^ and PL^pro^ assays were performed as previous reported [15, 23, 24, 37]. Briefly, the assay started with seeding 293T-ACE2 in 96-well, black, clear bottomed plate (Greiner, catalog No.655090) and incubating overnight to allow cells to reach 70–80% confluency. 50 ng of pLVX SARS-CoV-2 M^pro^ (or pcDNA3.1 SARS-CoV-2 PL^pro^) and 50 ng of pcDNA3 FlipGFP- M^pro^ (or pcDNA3 FlipGFP-PL^pro^) reporter plasmid was mixed with transfection reagent TranslT-293 (Mirus, catalog No. MIR 2700). The mixture was then transfected to each well according to manufacturer’s instructions. After 2.5-3 hours of incubation in 37°C, 1 μL of testing compound was added into each well directly and mixed by gentle plate shaking. 48 hours post transfection, fluorescence was quantified using SpectraMax iD3 plate reader (Molecular Devices) and images were taken using BZ-X800E fluorescence microscope (Keyence) in GFP and mCherry channels at 4X objective lens.

### Differential Scanning Fluorimetry (DSF).

The thermal shift binding assay (TSA) was carried out using a Thermo Fisher QuantStudio 5 Real-Time PCR system as described previously [10].

### Molecular docking.

Docking of PGG in SARS-CoV-2 PL^pro^ was performed using the Schrödinger Glide extra precision program. The X-ray crystal structure of SARS-CoV-2 PL^pro^ in complex with GRL0617 (PDB: 7JRN) was chosen for the docking. The gride box was centered on GRL0617. The docking poses were visualized using Pymol.

## Supplementary Material

Supplement 1

## Figures and Tables

**Fgiure 3:**
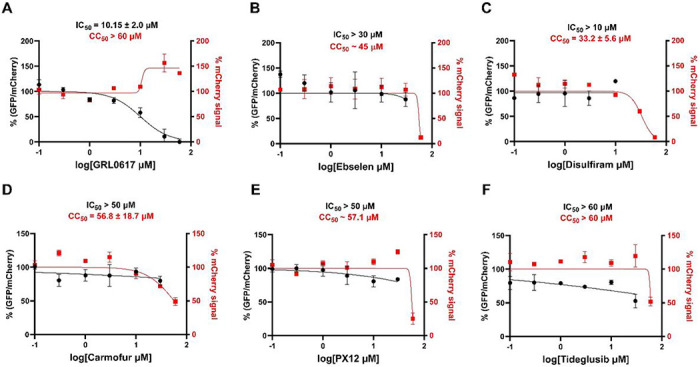
SARS-CoV-2 Flip-GFP PL^pro^ assay. GRL0617 (A) was included as a positive control. % (GFP/mCherry) ratio correlates with intracellular PL^pro^ activity, and % mCherry signal correlates with compound toxicity transfection efficiency. The results are mean ± standard deviation of two repeats.

**Table 1:** Validation and invalidation of SARS-CoV-2 M^pro^ and PL^pro^ inhibitors.

Compound	Reported SARS-CoV-2 M^pro^ inhibition IC_50_ (μM)	Reported SARS-CoV-2 PL^pro^ inhibition IC_50_ (μM)	Validation results IC_50_ (μM)
**Dieckol**	IC_50_ = 4.5 ± 0.4 (1 mM DTT) IC_50_ = 2.9 ± 0.2 (no DTT) Competitive inhibitor Ki = 3.3 μM [21] SPR K_D_= 0.22 μM	N.A.	**FRET assay**: M^pro^ IC_50_ > 20 (4 mM DTT) PL^pro^ IC_50_ > 20 (4 mM DTT) **Flip-GFP M^pro^ assay**: IC_50_ > 60 μM
**PGG**	SARS-CoV-2 IC_50_ = 3.66 ± 0.02 SARS-CoV IC_50_ = 6.89 ± 0.15 [22]	N.A.	**FRET assay**: M^pro^ IC_50_ > 20 (4 mM DTT) PL^pro^ IC_50_ = 3.90 ± 1.10 (4 mM DTT) **Thermal shift assay**: ΔT_m_ = 3.91 °C **Flip-GFP M^pro^ assay**: IC_50_ > 3 μM **Flip-GFP PL^pro^ assay**: IC_50_ > 3 μM
**Ebselen**	IC_50_ = 3.7 ± 2.4 (4 mM DTT) IC_50_ > 60 (4 mM DTT) [25]	IC_50_ = 10.3 ± 8.9 (4 mM DTT) IC_50_ > 60 (4 mM DTT) [25]	**Flip-GFP PL^pro^ assay**: IC_50_ > 30 μM
**Disulfiram**	IC_50_ = 2.1 ± 0.3 (4 mM DTT) IC_50_ > 60 (4 mM DTT) [25]	IC_50_ = 6.9 ± 4.2 (4 mM DTT) IC_50_ > 60 (4 mM DTT) [25]	**Flip-GFP PL^pro^ assay**: IC_50_ > 10 μM
**Carmofur**	IC_50_ = 0.2 ± 0.1 (4 mM DTT) IC_50_ = 28.2 ± 9.5 (4 mM DTT) [25]	IC_50_ = 0.7 ± 0.1 (4 mM DTT) IC_50_ > 60 (4 mM DTT) [25]	**Flip-GFP PL^pro^ assay**: IC_50_ > 50 μM
**PX-12**	IC_50_ = 0.9 ± 0.2 (4 mM DTT) IC_50_ > 60 (4 mM DTT) [25]	IC_50_ = 18.7 ± 2.6 (4 mM DTT) IC_50_ > 60 (4 mM DTT) [25]	**Flip-GFP PL^pro^ assay**: IC_50_ > 50 μM
**Tideglusib**	IC_50_ = 2.1 ± 0.3 (4 mM DTT) IC_50_ > 60 (4 mM DTT) [25]	IC_50_ = 7.1 ± 1.4 (4 mM DTT) IC_50_ = 30.4 ± 17.1 (4 mM DTT) [25]	**Flip-GFP PL^pro^ assay**: IC_50_ > 60 μM

N.A. = not available.
