# Supplementary figures and images for: Invalidation of dieckol and 1,2,3,4,6-pentagalloylglucose (PGG) as SARS-CoV-2 main protease inhibitors and the discovery of PGG as a papain-like protease inhibitor
Source: Res Sq. 2022 Mar 30:rs.3.rs-1490282. Preprint. [Version 1] doi: 10.21203/rs.3.rs-1490282/v1 (PMC8978949; doi:10.21203/rs.3.rs-1490282/v1)

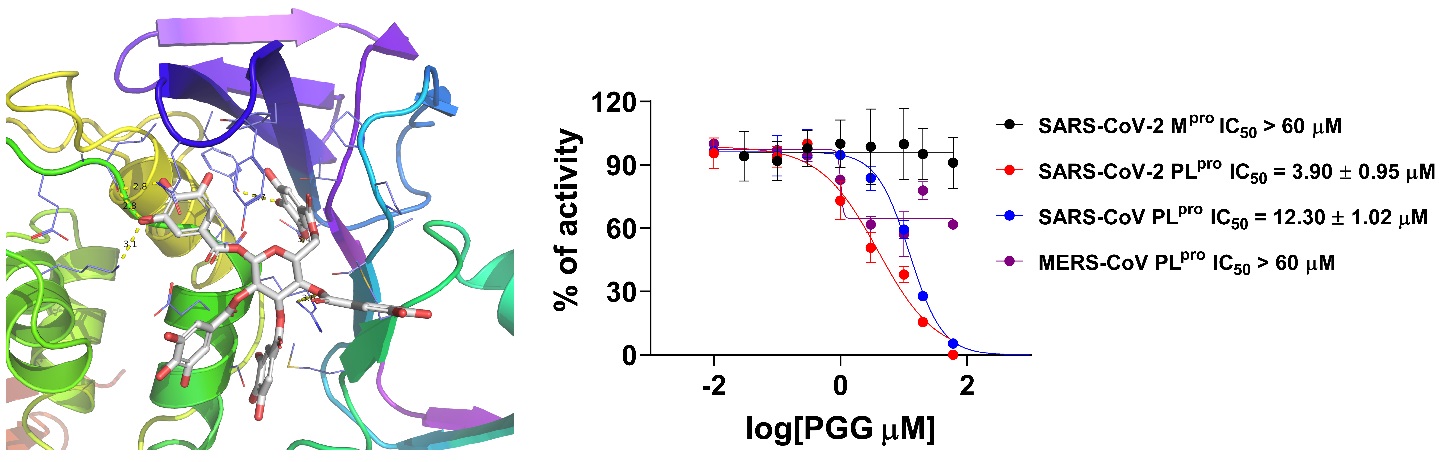

Supplement: Supplement 1 [file 4a949c52bbec6c6c00347757.jpg]
